# Good life in old age: Qualitative interviews about ageing with older adults with mild intellectual disability, prior to an educational intervention

**DOI:** 10.1177/17446295231213689

**Published:** 2023-11-11

**Authors:** Marianne Holmgren, Gerd Ahlström

**Affiliations:** Department of Health Sciences, Faculty of Medicine, 59568 Lund University, Sweden; Department of Health Sciences, Faculty of Medicine, 59568 Lund University, Sweden

**Keywords:** ageing, learning disability, older people, qualitative design, well-being

## Abstract

**Background:**

Knowledge about ageing from the perspective of people with intellectual disability is extremely scarce, which means a lack of evidence-based interventions for healthy ageing adjusted to their needs.

**Aim:**

To investigate how people with intellectual disability experience ageing, prior to an educational intervention.

**Methods:**

Twenty-six persons with mild intellectual disability, age 42-74 (mean 61.3) were interviewed and the text was analyzed qualitatively.

**Results:**

The main findings are reflected in the themes *Live for today – tomorrow you are old* and *Need of support to enable a meaningful ageing*. The participants avoided thinking about ageing, which they associated with retirement, loneliness and social isolation, increased need for help in everyday life, worsening health and death. Meaningful ageing meant continuance of leisure activities and working as long as possible.

**Conclusions:**

Interventions to prepare people with mild intellectual disability for healthy ageing must take into account these people’s loneliness.

## Introduction

In the Nordic countries, the life-conditions of people with intellectual disability have undergone significant changes over the past thirty years (Tøssebro et al., 2012). There has been movement from institutions to group homes or own homes with social service providing conditions that are as close as possible to those of the mainstream society; this in accordance with changes in policy and legislation (Tøssebro et al., 2012). This social integration together with medical development during the same period has contributed to increased longevity in people with intellectual disability in line with the same trend in the general population (Coppus, 2013; Dieckmann et al., 2015; Ng et al., 2017). A special case is that of people with Down’s syndrome explained by the progress in infant heart surgery, resulted in a decrease in mortality from 40.8% in 1973 to 4.8% in 2003 among children younger than one year (Englund et al., 2013). Despite this positive development, however, the gap in longevity for people with intellectual disability compared with the general population in high-income countries remains at about 20 years shorter (Glover et al., 2017; Robertson et al., 2021; O'Leary et al., 2018; Torr et al., 2010). As a consequence of greater longevity, people with intellectual disability may experience age-related morbidity (Haveman et al., 2011; McCarron et al., 2013; Torr et al., 2010; Bayen et al., 2018) with increasing need for support from the municipality (Bigby et al., 2011; Kåhlin, 2015; Ahlström et al., 2022). People with intellectual disability need to be seen as significantly more vulnerable people than others in society who are ageing (Bigby, 2002). Few people with intellectual disability have ever had paid employment, their social networks are limited (most often lacking a spouse or children), few have accumulated any assets before retirement, and most have been dependent on formal or informal support for everyday living. Many have been living in supported accommodation, such as group housing, or with their parents in adult age (Bigby, 2002). Older persons with intellectual disability can be lonely, isolated and passive because of insufficient staffing to support activities during daytime (Jormfeldt, 2016). The leisure activities are according to leaders in the disability service provided mostly in the evening (either by the municipalities or by voluntary organisations) when the older persons with intellectual disability generally are too tired to participate even if they would like to and therefore leisure activities become inaccessible for older persons with intellectual disability (Johansson et al., 2017). Older people with intellectual disability have also been reported to have less leisure activities than their younger counterparts (Dusseljee et al., 2011).

Fundamental in the UN Convention on the Rights of Persons with Disabilities (CRPD) (United Nations Human Rights, 2006) are autonomy, choice, independence, equality and participation. Several studies have shown that persons with intellectual disability themselves want to be a part of society to make their own decisions in everyday life (Bigby et al., 2004; Bigby and Knox, 2009; Jormfeldt, 2016; Björnsdóttir et al., 2015; Williams and Porter, 2017; Witsø and Hauger, 2020). In a focus groups study 68 older persons with intellectual disability discussed barriers to their participation in society and themselves suggested ways of reducing or removing them (Abbott and McConkey, 2006). This illustrates that it is both possible and essential to involve the older people themselves in the development of more appropriate provision of service (Abbott and McConkey, 2006).

Newberry and colleagues (Newberry et al., 2015) have shown that older people with intellectual disability have quite a wide range of thoughts about ageing. Seven participants (both men and women, aged 60-81) with mild intellectual disability were interviewed about their experience of the ageing process. The results shown themes such as ‘Relationships as central to enjoyment of life’ (having friends), ‘Powerlessness’ (restricted autonomy), ‘Needing a sense of purpose’ (having a job or activity to do) and ‘Making sense of getting older’ (having a purpose). This study found that there is a need to support people with intellectual disability in their transition to old age. McCausland and colleagues (McCausland et al., 2021) found that the interviewed carers (keyworkers and family) and older persons with mild, moderate or severe/profound intellectual disability (mean age 59.1) had both similar and different perspective on physical health, mental health and social care needs. The latter include accommodation needs, social and activation needs, employment needs and retirements needs. One implication from the findings on social care needs, particularly activation needs, was that it is essential to take into account the older person's own preferences (McCausland et al., 2021).

Now that people with intellectual disability are living into old age, it is of great importance to acquire knowledge of how these people themselves perceive the ageing process (Bigby et al., 2004). Furthermore, how education might help to prepare people with intellectual disability for their ageing is unknown. A literature search did not reveal to us any published research on education about ageing with older people with intellectual disability as participants. This being the case, an educational intervention for older people with intellectual disability that focuses on ageing can be seen as one effort to close the knowledge gap in this area. Against this background, the aim of the present study was to investigate how people with intellectual disability experience ageing, prior to an educational intervention.

## Methods

### Design

The study had an exploratory qualitative design based on qualitative interviews with persons with intellectual disability in four towns in Sweden in which an educational intervention entitled “Good life in old age” was to be implemented.

### Educational intervention

The educational intervention “Good life in old age” is founded on study circles, with 25-30 weekly meetings a year for two years. The goal is that persons with intellectual disability will become senior experts in ageing issues, able to share their knowledge with politicians, civil servants or journalists and thereby foster the development of better social services and healthcare for people with intellectual disability.

The intervention project was initiated by an experienced former administrator for ageing issues at the National Association for Persons with Intellectual Disability (FUB). The project leader developed the content of the education together with persons with intellectual disabilities from local associations of FUB. This was performed in group meetings and through individual interviews with persons other than those in this study, where they designated what they perceived as important content. A project group consisting of the project leader and two experienced members were responsible for implementation of a study guide and prepared a schedule of 25-30 weekly meetings a year in collaboration with appointed study circle leaders. All of them had special competence within the realm of intellectual disability. The overall themes for the study circles were ageing, accommodation, activities and opportunity to influence. In addition to teaching on the themes, the programme included several study visits, visits by experts, group discussions and continuous follow-up of the meetings. This educational intervention will be evaluated in a future study and then described in more detail.

### Sampling procedures and participants

Eligible for participation were persons with mild intellectual disability who lived in or close to one of the four towns spread over Sweden and were well-known to study circle leaders from the Adult Educational Association (*Vuxenskolan*) and the local branch of the Swedish National Association for People with Intellectual Disability (*FUB*). Forty-nine persons with mild intellectual disability were invited to participate in the educational intervention and 29 accepted the invitation. In the next step, these persons were invited to participate in this interview study and three of them refused (two females and one male). Thus the total number of participants in the study was 26: 17 female and 9 male, aged 42‒74. Twelve were working, three were of working age but were not in the labour market, and 11 were retired. Eleven lived in a flat of their own, one lived in the parents’ house and 14 lived in group housing or service housing. (Group housing: each person has a small flat, living together with 5-8 other people in the same residential area. Service housing: each person has a small flat with access to supporting staff around the clock). All except one receive help in handling their finances from a trustee or a family member, and 14 have access to support around the clock.

### Interviews

Photos in large format related to the themes of the interview questions were presented on a computer screen to increase the validity of the answers. These photos were of older people in everyday situations relevant to the interview areas. Twenty-one interviews were face-to-face and five were conducted via computer (Zoom) during the period August to November 2021. The interviews (in which the older persons participated independently) lasted between 23 and 105 minutes (median 49 min; mean 51 min). Each of the two authors interviewed the persons with intellectual disability in two towns and field notes were written when the interview was finished. The interview guide was semi-structured and was designed to capture the participants’ views on the themes to be addressed in the study circles. The guide included the following questions:

#### Ageing

1) What does it mean to become older?
2) What is retirement? (Follow-up questions whether the person was retired or not).

#### Activities for older adult persons

3) What do older people do during the day?

#### Accommodation

4) Where is good to live when you are older?
5) How would you like to live?

#### The opportunity to influence

6) Who decides what you do during the day? (with regard to activities).
7) Who decided where you were to live?
8) Have you met someone who decides how things are to be for you? (a politician or a manager within the disability service).

An additional question concerned expectations concerning the study circles:

9) What is good about joining a study circle like this? (for you and others).

All interviews were digitally recorded and transcribed verbatim.

### Qualitative analysis

The transcribed interviews were submitted to qualitative content analysis by both authors. The field notes were integrated in the analysis to facilitate the interpretations of the texts. Qualitative content analysis focuses on subject and context as well as variation of meanings between parts of the text (Graneheim and Lundman, 2004; Elo and Kyngäs, 2008). Initially, this analysis was performed by the first author. The transcribed text from the interviews was read through several times to obtain an overall picture of the content and thereafter meaning units were identified, these being then condensed and labelled as codes. The first level of codes, named sub-categories, were generated through identification of similarities and differences in the text. These manifest sub-categories were based on a prudent interpretation. Next, the sub-categories with similar content were abstracted and merged into latent main categories (Graneheim et al., 2017).

In the next phase, the second author read all of the interviews and the identified meaning units and performed an independent critical review of the suggested codes, sub-categories and main categories.

In the third phase, the whole content of the sub-categories and the main categories was interpreted in discussions between the two authors, based on a continuous iterative process that went back and forth between parts and the whole until a shared understanding of the text emerged. These interpretations resulted in the establishment of two themes. Sub-categories, categories and themes are described in a theoretical hierarchy, giving a “thick description” (Graneheim et al., 2017). NVIVO software (Release 1.6.1) was used as a facilitating tool through the whole coding process and sorted the accompanying text to the codes (Edhlund and McDougall, 2020). The names attached to the quotations in the Results section are fictitious.

### Ethics

The process of inviting participation in the study was conducted in two steps to ensure that consent would be properly informed. First, the invitation to participate in the study was proffered by the designated study circle leader, who knew that the person had mild intellectual disability. The study was presented by the study circle leaders in the particular town at an information meeting for presumptive participants in group or individually by personal contact. A pre-recorded video was used where a person with mild intellectual disability was seen interviewing the researcher. The video contained information about the aim of the research and execution of the study. The persons with intellectual disability who were interested in participating in the study were provided with easy-reading written information, and they had the opportunity to ask questions about the study. The second step was that, before the individual interviews started, the researchers repeated the information about the study and emphasised that participation was voluntary and that the interview could be cancelled or broken off at any time. Every efforts was made to make sure that the persons with intellectual disability understood the information. All presumptive participants agreed to be interviewed and written informed consent was signed by each participant before the interview started. The Swedish Ethics Review Authority (*Etikprövningsmyndigheten)* approved this study (Dnr 2021-01539).

## Results

The results are presented as two themes, five main categories and 2–3 sub-categories in each main category (Table 1).

**Table 1.** Overview of themes, categories and sub-categories.

Themes	Main categories
	Sub-categories
Live for today, tomorrow you are old	Disregard of ageing
	Ageing is non-existent
	Living life now
	Continue working
Need of support to enable a meaningful ageing	Grasping the transition to becoming old
	Ageing is becoming wiser
	Worrying about health problems
	Looking upon ageing in terms of dementia
	Difficulty of anticipating retirement
	Starting-shot for ageing
	Freedom to decide
	Loneliness after retirement
	Desire for enjoyable activities in old age
	Looking at possibilities of activities
	Inability to take the initiative
	Expectation of being able to decide on accommodation
	Wish to stay put with more support
	Value of living in social community

The participants’ described their own ageing at different levels of awareness. On the one hand, they avoided thinking about ageing. They did not see themselves as being engaged in an ageing process, independently of what their own age was. On the other hand, they saw ageing as a natural part of life, meaning that they wanted to live their life here and now. A few of them expressed also a desire to become prepared for old age. Not being alone in old age was highlighted as something very important, as was participation in meaningful activities in company with other people. This was also reflected in the answers to the final interview question about expectations regarding the upcoming educational intervention. They stressed that they wanted to meet and socialise with others while the need for new knowledge about ageing was less strongly emphasised.

## Live for today, tomorrow you are old

### Disregard of ageing

#### Ageing is non-existent

Several participants had difficulty in expressing what ageing is, they had not even thought about it. They avoid talking about the subject, as if it were a non-existent process for themselves. They did not perceive themselves as ageing or as old people, independently of their own age.

*Interviewer: What does it mean to be an older person?*

*Participant: I’ve never thought about it, not even once.*

*I: What if you think about it now?*

*P: Think about it now? There’s not much I can think about it. I don’t know how to answer the question, it’s not a thing I’ve ever given any attention*. (George)

Thus, when the participants talked about older adult people, they were not talking about themselves, they were talking about other people. Older people meant those who were older than they were, aged anything between 60 and 100. During the interview, the participants were shown photos of older people, and they spoke of wrinkles and white hair as determining whether they thought the people were older adults or not. They did not see themselves as having these signs of ageing.

*I*: *And what do you think makes her look old, when you look at her?*

*P: She’s got white hair, white hair, and she’s sort of sunken in the face*. (Emily)

#### Living life now

The participants said that they lived their life now, they did not think of the process of ageing at all, they wanted to have fun here and now, taking one day at a time. They were very easy-going, not thinking ahead at all about what might happen when they got older. Ageing was far away and nothing to worry about right now.

*What I say is, take one day at a time. I always say that, because you mustn’t worry too much, if you’re going to age you’re going to age.* (Elizabeth)

#### Continue working

The participants who were still working said that their target was to continue doing so. They thought hardly at all about their retirement and did not want to retire because they enjoyed working. They said that they wanted to go on working as long as they were in good health and as long as they were allowed to work.

*I: You said you were 61. Have you ever thought about retirement?*

*P: No, I want to go on working. I don’t want to retire until I’m 65. I want to go on, as long as I’m up to it, want to keep working. Until my body says no*. (Martin)

In addition, there were some participants who shared their concerns about forthcoming retirement. They imagined that life would be both lonely and boring after retirement.

*I’d like to feel prepared before I retire that it’s going to be good, and that I won’t be too lonely. I’d like to feel sure before it's time*. (Emma)

## Need of support to enable a meaningful ageing

### Grasping the transition to becoming old

#### Ageing is becoming wiser

As opposed to those who had difficulty in expressing what ageing is, there were participants who described ageing as a natural part of life. These participants had the ability to reflect on aging. They said that with experience came wisdom, and they did indeed feel that they had become wiser over the years. Such wisdom could involve pondering. The participants spoke of how often they saw ageing people, both those they knew and those they did not know, sitting pondering.

*I*: *What does it mean to become older?*

*P: I think it’s good, getting older, I think it’s good.*

*I: What’s good about getting older?*

*P: Well, I suppose you’ve grown wiser with the years. Growing wiser with the years, I don’t know, I suppose it’s something like that.* (Jack)

Questions about earlier ageing for those with intellectual disability, especially for those with Down’s syndrome, were raised by the participants during the interviews. There was expressed as a general worry that this could also affect themselves. It emerged that ageing was not a topic that care staff or anyone else had talked to the participants about before.

#### Worrying about health problems

The participants associated ageing with ailments, illnesses and other health problems and with increased need for help in everyday life. There were expressions of concern about ending up in a wheelchair. There was also a fear of falling and some participants would not dare to walk without a walker.

*…. I can't say exactly what it is but I got it into my head that I was going to fall all the time and trip over things, and that's why I've felt that maybe I should try and see. And now I don't dare walk without a rollator, which I think is a great pity*. (Li)

They talked also about difficulties arising from such things as lack of energy, slower movement and difficulty in balancing.

*Oh dear [laughs], what shall I say? A few more aches and pains — a few more aches and pains than when you were young, for instance. And you perhaps don’t function the same way as you did when you were younger. And, well, what more can I say?* (Joe)

When the various physical limitations appeared, the need of aids such as a rollator or cane increased. Even though the participants perceived the use of aids as belonging to ageing, they knew that also younger people use aids because of disability. The need of aids increased when balance got worse, and this worsening of balance was a sign of being older and in need of help.

*You perhaps get a bit stiffer and — oh dear, yes — your sight gets worse, that can happen. Yes, I’d say there are an awful lot of different things that can happen to your body — that actually do happen, I suppose.* (Samantha)

The topic of death came up when the participants talked about ageing. They shared their thoughts about anxiety, pain and loneliness in the face of death. One question that was raised was: “Do you think that there will be hospice care for people with intellectual disability too?” Some participants said that they did not want to die alone and that they needed to prepare themselves. One person spoke of meeting loved-ones after death, with use of a reference to a celebrated Swedish author of books for children:

*P: One doesn’t know where one’s going to go in the end, of course, but I’ve got a bit of a notion of it.*

*I: No, one doesn’t know.*

*P: I’ve got a notion of it like Astrid Lindgren in The Lionheart Brothers.* (Michael)

#### Looking upon ageing in terms of dementia

Dementia belongs to old age. When the participants talked about age-related diseases, they mainly talked about dementia, where the person forgot things and became disorientated. Some of them had experience of it within their own families. They spoke of the anxiety and difficulties in connections with their partner’s or a parent’s suffering from dementia.

*He took retirement and that was fine but then he went out on his bike and couldn’t find his way back. It made me so anxious — in the end it was too much.* (Jessica)

*You perhaps get stricken with dementia, for instance. You start forgetting things — though that doesn’t have to be a sign of dementia, of course. You begin to lose your bearings, find it harder to find your way around, even to places you’ve been before. That’s how it can be.* (Jacob)

### Difficulty of anticipating retirement

#### Starting-shot for ageing

Retirement was looked upon as a starting-shot for ageing by those who were retired. However, they remembered the fear of retirement beforehand. They had had no idea what awaited them and leaving their job was leaving security and friends. It took some time for them to become accustomed to their retirement. Even when they finally felt comfortable with it there remained a certain insecurity concerning money, loneliness and access to activities.

*Well, I suppose it was more a question of my financial situation, it was going to get worse. Another thing was that it’d be terribly lonely and be hard for me to find things to occupy my time.* (Jacob)

#### Freedom to decide

The participants who were retired said that it was nice to be able to decide how they would spend their days, gave them a feeling of freedom. However, it was not a feeling that occurred immediately after retirement, it took a while for it to emerge. Freedom was commonly expressed in the choice of activities, it was liberating to be able to decide for yourself what you were going to do during the day.

..*.but now I can say no to the things that I don't think are fun and only do the things that I think are fun. It’s an advantage of getting older and retiring that you can choose more, decide for yourself, decide more for yourself what you want to do and not do*. (Jacob)

Despite their freedom, however, the participants were missing their jobs and had several stories to tell about the jobs they had had over the years. In addition they expressed thoughts about what was the right time to retire. They considered both 65 and 67 as appropriate ages for retirement. It should be up to them to decide. They said, too, that the same rules should be applied for those with intellectual disability as for those without intellectual disability.

*Just as you others have the right to put off retirement until you’re 67, so should we have.* (Victoria)

#### Loneliness after retirement

The participants said that they felt lonely after retirement. They had feared this even before retirement. They missed their former colleagues and in many cases had lost their social network because they had no friends outside of their jobs. They needed a new forum for socialisation. There should be help in preparing for retirement to prevent loneliness.

*I: You said, didn’t you, that the people you came into contact with at work disappeared when you retired. Were they pals of yours?*

*P: Yes, you could say they were. Mm. And then there were those that retired before I did. But, well, we didn’t see so much of each other in private life, it was just at work.*

*I: I see. But did you have any pals outside work?*

*P: Unfortunately no.*

*I: So your job was important to you?*

*P: Yes.* (Margaret)

The vulnerability of retired people with intellectual disability was highlighted by the corona pandemic. The interviews took place directly after society had opened up again after shutdown and therefore the pandemic was fresh in the memories of the participants. They described a pervasive loneliness and boredom in association with the interruption of activities caused by the pandemic. The importance of activities became clear when every activity was cancelled.

### Desire for enjoyable activities in old age

#### Looking at possibilities of activities

With retirement, leisure activities became more important. The participants continued with activities they had been engaging in before, at the same time as there were other ones they wanted to try. Activities mentioned (some engaged in before, some not) included the following: getting together with friends, watching television, playing games, going on trips, playing boules, going dancing, participating in study circles, doing needle-work and going out for a meal.

*Boules, that’s an idea. A lot of people play boules, Mum and Dad did [laughs a little]. Then there’s bingo, and there’s entertainment.... And then of course there are walks, where those of us who use rollators are included..... We can start with a rollator rally [laughs a little] and go on a quiz walk. I read that some group of people with rollators had said there should be rollator rallies.* (Emma)

#### Inability to take the initiative

The participants said that they found it difficult to take the initiative when it came to leisure activities.

*The problem as I see it, when you have an intellectual disability, is that you most often find it difficult to take the initiative, and you most often have so few friends, so few people you can connect with — it’s mostly the people who live where you do, but of course most of them have jobs to go to*. (Jacob)

Because of the difficulty they themselves had in taking the initiative with regard to activities, the participants looked to the staff for suggestions. However, they felt that the staff were not interested in helping them take the initiative and performing the activities together with them.

The participants said that they looked forward to taking part in the forthcoming study circle because it was in their interest to learn more about ageing. Their main reason for taking part, however, was to have company. It would give them something to do once a week, having coffee with other people and chatting.

### Expectation of being able to decide on accommodation

#### Wish to stay put with more support

The participants were not quite sure where they wanted to live when they got older. It appears that their current accommodation influenced their view of future accommodation. Those who already live in group housing or service housing for people with intellectual disability are likely to continue living there. Those who live in a flat of their own are likely to stay put with increased support and service from the municipality.

*Now the staff have said they’ll come and help me more. So I don’t need to go to an old people’s home.... They’ve promised to come and help me more and more. But it can be a good thing to die in hospital, get help there, rather than having them come up here to the third floor with a stretcher*. (Michael)

Participants expressed an insecurity about the possibility of remaining in their own flat when they needed more help. It became clear that they had doubts as to whether the home-help service could provide what special housing could. They would not want to stay put if they did not get enough help.

*No, I wouldn’t want to stay put with home help.... No, I wouldn’t, because I know they count the minutes, and I want to live where they don’t count the minutes. I’d like my last place to live to be a home for older people, because you know you’ll have company there. But now it’s so difficult to get in. It used to be easier to get into an older people’s home. A lot of people say they’d rather go there than stay put in their own flat.* (Emma)

#### Value of living in social community

Those who wanted to live in group housing wanted to live together with other older people, who could be expected to have interests similar to their own. They did not want to live in group housing where there were younger people. Furthermore, it was important to them to have a small flat of their own in a shared facility where they could be on their own and could come and go as they pleased. At the same time they did not want to feel alone. And it was important that they should be able to participate in the decision-making in respect of their living-situation. Living in the centre of town was on their wish list. Furthermore they wanted to have access to staff who were caring and showed them respect. However, if they became stricken with dementia, special housing for older people with dementia would be the best.

*P: No, I’ve told him once before, I want to get away from here, to one of those flats where older people live. I want to live with people the same age as me. There are too many young people here.*

*I: Yes.*

*P: There are too many young people here. They’re 50, some of them 49. They’re young people, or anyway they’re not as old as me.* (Peggy)

## Discussion

In this study, the participants’ statements gave rise to two themes. The first of them, “Live for today, tomorrow you are old” speaks for the avoidance of the subject of ageing. Best is to live in the present and not think about ageing, and to continue working as long as you can. On the other hand the second theme, “Need of support to enable a meaningful ageing”, speaks for the future. The participants were uncertain as to how to prepare for ageing but anticipated being able to do what they wanted. Meaningful ageing included activities after retirement, with a need of support from the staff. The importance of activities was made evident when they were all cancelled during the corona pandemic. Here the vulnerability of retired people with intellectual disability was highlighted. Meaningful ageing also included having access to the sort of accommodation that suited you.

The participants’ avoidance of the subject of ageing was manifested in that they had not given it any thought, and those who were working had no plans for retirement. Nor did those who were working have any clear idea of what awaited them after retirement. At the same time, they perceived retirement as a critical event, a clear starting-shot for aging. Schepens and colleagues (Schepens et al., 2019), in their systematic review which concerned people with intellectual disability ≥50 years of age, reached similar findings. Few of the working people with intellectual disability had a clear idea of what awaited them after retirement and they feared the loss of companions. Avoidance means attempting to change the nature and frequency of unpleasant thoughts, emotions and sensations with the goal of not being in contact with them. This way of coping with negative life-events has been shown to be related to anxious and depressive symptomatology in older people and in general (Fernández-Fernández et al., 2020; Ferguson et al., 2017; Knight, 2020; Rotenberg et al., 2020). However, avoidance in persons with intellectual disability is not only a question of evasion and escape, it needs also to be understood in relation to each person’s cognitive ability and limitations. Intellectual disability means significantly reduced ability to understand new or complex information and to learn and apply new skills (WHO, 2021). One way to facilitate healthy ageing and well-being in persons with intellectual disability is through an adapted gerotranscendence approach. This approach means that the human being is seen as continuing to develop even in old age and that ageing should therefore be seen as a lifelong process which, in the best case, leads to new perspectives and an increase in experience (Tornstam, 1996; Wadensten, 2005; Jewell, 2014; Wadensten, 2010). In our study, the participants’ talk of attaining wisdom and sitting pondering can be a sign of gerotranscendence. They looked on older people, both those they knew and those they did not know, as sitting pondering. Gerotranscendence is facilitated for older persons with intellectual disability when they gain more knowledge about the meaning of ageing and are given plenty of time to reflect and talk things over with supportive contact persons or staff in the community service. Based on this finding, it would be rewarding to evaluate an implementation of the gerotranscendence perspective in social services for older people with intellectual disabilities, as has been done for older people in nursing homes (Wadensten, 2010).

Asked what they thought about ageing, the participants spoke of increased health problems with increasing need for help in everyday life. They mentioned such age-related health problems as lack of energy, slower movement and difficulty with balance. Dementia was the main age-related disease they talked about: it made you forget things and made you disorientated and finally it led to your death. Their worrying about poorer health is in line with their actual vulnerability. Besides the lifelong limitations in reasoning, learning, problem-solving, and adaptive behaviour, older people with intellectual disability have a greater risk of multimorbidity (McCarron et al., 2013), medication use (O'Dwyer et al., 2016; Axmon et al., 2017), negative health outcomes and mortality than people in the general population of the same age and sex (Ng et al., 2017). Furthermore, research has shown that they are more likely to have more inpatient and unplanned visits in both somatic and psychiatric healthcare as well as readmission within 30 days of discharge, compared to people of similar ages and of the same sex in the general population (Ahlström et al., 2020; Axmon et al., 2019; Sandberg et al., 2016). In sum, older people with intellectual disability are especially vulnerable not only because of their cognitive and physical limitations but also because of their poor socio-economic resources, their lack of a social network and their lower level of participation in society (Bigby, 2008; Engeland et al., 2021).

In line with our findings, leisure activities were also highlighted by Buys and colleagues (Buys et al., 2008), who found that people with intellectual disability aged 52-80 wanted to ‘keep on keeping on’, which is to say keep doing these activities in the future. When it comes to active and healthy ageing, it is important that staff provide encouragement and close support, particularly for those who are making the transition from work to retirement. Also, Schepens and colleagues (Schepens et al., 2019) found in their review that people with intellectual disability express a need of enjoyable leisure activities and of opportunities to do things with staff, friends and family, which is in line with our own findings even though family is seldom mentioned in our study.

The basic right to good life-conditions for people with extensive disabilities is set out in the Act on Support and Services for Persons with Certain Disabilities (LSS), (SFS 1993:387). The law is based on the CRPD (United Nations Human Rights, 2006) and emphasises self-determination and influence, which are often used overlappingly in research contexts. In this study self-determination was found to be important for the participants especially in respect of the choice of accommodation when they got older and had increased need of care. They did not have the same wishes regarding accommodation but they all had the same wish to participate in the decision-making. Staff should support them in this (Bigby et al., 2011). However, such support has proven to be a challenge. Browning and colleagues (Browning et al., 2021) have shown how staff differ when it comes to offering support. Some are more vigorous than others; some use one support strategy, others use another. The support needs to be an iterative process to produce effective decision-making. Furthermore it has to be geared to the particular person’s unique situation.

A feeling of loneliness was associated by the participants with four separate situations. First and foremost there was retirement, involving loss of the community with fellow-workers. Second there was the corona pandemic, when all leisure activities were shut down for a little over two years. Without leisure activities the participants lost their social network, lost much of their contact with their friends. Third there was the situation with regard to accommodation. They did not want to be alone if they became ill and in need of care, they wanted in this case to move to special housing with others who had interests similar to their own. Fourth there was the situation where one was facing death. The prevalent occurrence of loneliness among persons with intellectual disability is confirmed by a systematic review which showed an average prevalence of 44.74% (Alexandra et al., 2018), as compared with 10.5% in the general population (Beutel et al., 2017). Loneliness is commonly defined as a painful experience of absence of social contact, absence of belongingness (Beutel et al., 2017; Alexandra et al., 2018). The suffering is greater among those living alone, without a partner and without children (Beutel et al., 2017). This means that it is commonly greater among older people with intellectual disability. The social network of older people commonly consists of fellow-workers (before pension) and paid staff. Longitudinal research has identified loneliness as a vulnerability factor for negative mental health outcomes in the general population. Beutel and colleagues (Beutel et al., 2017) confirmed that loneliness was associated with depression, generalised anxiety and thoughts of suicide, as also with more frequent visits to physicians. Despite the need of reducing loneliness among older people with intellectual disability, the current literature shows shortage of proposals regarding interventions to support such people in developing and maintaining social relationships (Alexandra et al., 2018; Scott and Havercamp, 2018). Researchers, policy-makers and service providers need to develop and evaluate such interventions.

The participants said that they wanted to be prepared for old age. In the Nordic countries, governments have in recent years taken various initiatives dealing with active and healthy ageing that are being implemented at various levels of the welfare services. Also, the WHO domains of the Global Age-friendly Cities Guide, developed to identify and address barriers to the well-being and participation of older people, have been adopted in a network of countries including the Nordic ones (Nordic Welfare Centre, 2022; WHO, 2022). However, the literature on policy neglects to take account of the life-conditions of older people with intellectual disability, which means that this vulnerable group continue to be excluded from participation in society. The findings of the present study emphasise the need for a more comprehensive understanding of active and healthy ageing, ensuring that proper account is taken of the perspective of older persons with intellectual disability in policy reports, in research and within the social services and healthcare.

### Methodological considerations

It is important to consider some methodological issues in respect of this study. Despite the care with which we devised and conducted the procedure of inviting participation in the study (with video film about the project, oral information by the study leaders), a possible weakness of this procedure was that the study circle leaders delegated the task of providing information about the study to staff who knew the person much better. That means that it is unknown whether everyone used the computer-based link to the pre-recorded video. However, later when the researcher repeated the information about the study and invited questions before the giving of written consent, it appeared that everyone was well-informed about the study. This shows that the person who knew the presumptive participant used individualised communication prior to obtaining informed consent to participate in the study (Cambridge and Forrester-Jones, 2003).

The participants varied in their ability to express their thoughts and feelings in the interviews, which is reflected by the different character of the quotes. Therefore, the principle of no-harm had to be ever-present in the minds of the researchers (Hall, 2013; The World Medical Association, 2013). The risk of harm to the person's integrity, privacy and right to self-determination, was minimised through keeping questions at the right level for the person, listening properly to what the person said and being sensitive to their wishes even if expressed in unusual words or in other ways such as through body language (Cambridge and Forrester-Jones, 2003). We have experience and expertise in the field and furthermore had the advantage of being able to consult a very experienced special education teacher and researcher (Cecilia Olsson) who has expert knowledge of how to interview persons with intellectual disability. The utmost effort was made to establish the best form of communication and to conduct the interview with full respect for the person’s integrity. Being listened to with respect boosts the confidence of vulnerable persons and gives them a greater sense of belonging in society. This is particularly important for a group of people who do not otherwise have forums to express their views. It can be seen as a question of justice.

In addition to individualised (person-centred) communication in the interviews, we used photographs shown on a laptop. This is one way that is recommended in the paper by Cambridge and Forrester-Jones (Cambridge and Forrester-Jones, 2003) to facilitate communication in the interviewing. The participants younger than retirement age found it in general harder to express thoughts about ageing, and this needs to be taken into consideration in future studies.

To achieve credibility, it is essential to find participants who have experience of the phenomenon under study and can talk about it (Graneheim and Lundman, 2004). The participants varied in their capacity for oral communication, which was a challenge in the analysis of the interviews. Therefore both authors read all the interviews and were engaged in all phases of the analysis, each maintaining a critical attitude to their own and the other researcher’s interpretation of the interview texts. These texts, as also memos from the open coding and field notes, were subjected to interpretive content analysis with a focus on identifying the connecting thread discernible in categories and themes. Trustworthiness was fulfilled through the process of organising data and iterative interpretation with a progressively increasing understanding of the participants’ voices (Graneheim and Lundman, 2004). However, it is important to keep in mind that the results of this study apply to people with *mild* intellectual disability and that the transferability of these results to people with moderate or profound intellectual disability needs to be very cautiously weighed. If people with more severe ID had described their ageing, they might have described it differently. Therefore, there is an urgent need to initiate studies similar to this one, using alternative communication tools to give a voice to people with moderate or severe intellectual disability and low verbal ability. Most of the participants were receiving support from social services and only one was living in the parents’ house. This means that the findings are not applicable to those who live with their family and receive care of aged parents. Much more research on ageing is needed in the area of intellectual disability.

## Conclusions and implications

The attitude to ageing is expressed in the two overriding themes “Live for today, tomorrow you are old” and “Need of support to enable a meaningful ageing”. Living life here and now was connected with avoiding thoughts about ageing. Those participants who did have something to say about ageing perceived it as starting with retirement and as being associated with health problems, need for help in everyday life and dying. Ageing was mostly related to worries about loneliness and boredom due to social isolation after retirement. The positive side of ageing was becoming wise, being able to keep on with leisure activities in company with other people and being able to go on working. Participants stressed the value for them of meeting and socialising with others. The implications from the findings of this study highlight the urgent need for preparing people with mild intellectual disability for active and healthy ageing before retirement. These people’s pervasive feeling of loneliness needs to be taken into account when it comes to support within the disability services. One way to develop support is to apply gerotransendence perspective, which means allowing the voicing of reflections about ageing and also the discussion of it. The staff require knowledge to implement this perspective in a proper way. In addition, education about ageing in older people with intellectual disability can increase these people’s participation in the ongoing changes in society and enable professionals, researchers and policy-makers to understand these people’s particular needs. The educational intervention, which is at the planning stage, has also the goal that persons with intellectual disability shall become senior experts in ageing issues, able to share their special knowledge with politicians, civil servants and journalists. This dissemination of such knowledge can reduce stigmatisation and discrimination, which can mean better social services and healthcare for people with intellectual disability.
